# Tumor Lysis Syndrome in Chronic Lymphocytic Leukemia with Novel Targeted Agents

**DOI:** 10.1634/theoncologist.2017-0055

**Published:** 2017-08-29

**Authors:** Bruce D. Cheson, Sari Heitner Enschede, Elisa Cerri, Monali Desai, Jalaja Potluri, Nicole Lamanna, Constantine Tam

**Affiliations:** ^a^ Georgetown University Hospital, Lombardi Comprehensive Cancer Center, Washington, DC, USA; ^b^ AbbVie, North Chicago, Illinois, USA; ^c^ Columbia University Medical Center, New York, New York, USA; ^d^ St Vincent's Hospital, Melbourne, Australia; ^e^ Peter MacCallum Cancer Centre, Melbourne, Australia; ^f^ University of Melbourne, Melbourne, Australia

**Keywords:** Chronic lymphocytic leukemia, Tumor lysis syndrome, Targeted cancer agents, Hematologic malignancies

## Abstract

Tumor lysis syndrome (TLS) is an uncommon but potentially life‐threatening complication associated with the treatment of some cancers. If left untreated, TLS may result in acute renal failure, cardiac dysrhythmia, neurologic complications, seizures, or death. Tumor lysis syndrome is most commonly observed in patients with hematologic malignancies with a high proliferation rate undergoing treatment with very effective therapies. In chronic lymphocytic leukemia (CLL), historically, TLS has been observed less often, owing to a low proliferation rate and slow response to chemotherapy. New targeted therapies have recently been approved in the treatment of CLL, including the oral kinase inhibitors, idelalisib and ibrutinib, and the B‐cell lymphoma‐2 protein inhibitor, venetoclax. Several others are also under development, and combination strategies of these agents are being explored. This review examines the diagnosis, prevention, and management of TLS and summarizes the TLS experience in CLL clinical trials with newer targeted agents. Overall, the risk of TLS is small, but the consequences may be fatal; therefore, patients should be monitored carefully. Therapies capable of eliciting rapid response and combination regimens are increasingly being evaluated for treatment of CLL, which may pose a higher risk of TLS. For optimal management, patients at risk for TLS require prophylaxis and close monitoring with appropriate tests and appropriate management to correct laboratory abnormalities, which allows for safe and effective disease control.

**Implications for Practice.:**

Tumor lysis syndrome (TLS) is a potentially fatal condition observed with hematologic malignancies, caused by release of cellular components in the bloodstream from rapidly dying tumor cells. The frequency and severity of TLS is partly dependent upon the biology of the disease and type of therapy administered. Novel targeted agents highly effective at inducing rapid cell death in chronic lymphocytic leukemia (CLL) may pose a risk for TLS in patients with tumors characterized by rapid growth, high tumor burden, and/or high sensitivity to treatment. In this review, prevention strategies and management of patients with CLL who develop TLS are described.

## Abstract (precis)

Tumor lysis syndrome is an uncommon but potentially life‐threatening complication associated with the treatment of some cancers. In this review, prevention strategies and management of patients with chronic lymphocytic leukemia who develop tumor lysis syndrome are described.

## Introduction

Tumor lysis syndrome (TLS) is a well‐recognized condition caused by the abrupt release of cellular components into the bloodstream after massive lysis of malignant cells, which can be potentially life threatening [1], [2]. The release of large amounts of potassium, phosphorus, and nucleic acids overwhelms normal homeostatic mechanisms, resulting in hyperkalemia, hyperphosphatemia, hyperuricemia, and secondary hypocalcemia [3], [4]. Although TLS most often occurs during the first cycle of therapy, it may also occur later in the course of treatment. Left untreated, TLS can lead to acute renal failure, cardiac dysrhythmia, neurologic complications, and seizures [5], [6]. Tumor lysis syndrome most commonly occurs during cytotoxic chemotherapy, although biological agents such as anti‐CD20 monoclonal antibodies have also been shown to induce TLS [6], [7]. Occasionally, TLS may occur spontaneously in patients whose tumors have a high proliferative rate, such as diffuse large B‐cell lymphoma, acute lymphoblastic leukemia, or Burkitt lymphoma, but it is most commonly observed after the initiation of chemotherapy [6], [8]. An increased incidence of TLS has been observed with the advent of increasingly effective targeted therapies for a wide range of tumor types [3]. Although TLS was historically observed in patients with chronic lymphocytic leukemia (CLL) treated with chemotherapy [9], it was uncommon and not typically severe. The introduction of targeted agents that can cause rapid tumor reduction has increased the risk for TLS in these patients [10].

## Types of TLS and Diagnosis

Tumor lysis syndrome may occur either as laboratory TLS or clinical TLS. Laboratory TLS is by far the more common, and is defined as the occurrence within a 24‐hour period of two or more electrolyte derangements (i.e., hyperkalemia, hyperphosphatemia, hypocalcemia, hyperuricemia) in an asymptomatic patient from 3 days before to 7 days after treatment [11]. Clinical TLS is a rapid and extreme change in serum electrolytes, consistent with laboratory TLS with additional clinical complications and the potential to lead to clinical sequelae, which can develop suddenly in conjunction with cancer treatment, and represents a medical emergency requiring aggressive intervention [10]. Clinical complications may include nausea, vomiting, lethargy, edema, renal failure, congestive heart failure, and potentially sudden death. Less frequently observed is subacute TLS, which is characterized by gradual changes in laboratory values [10].

Although TLS was historically observed in patients with chronic lymphocytic leukemia (CLL) treated with chemotherapy, it was uncommon and not typically severe. The introduction of targeted agents that can cause rapid tumor reduction has increased the risk for TLS in these patients.

Two sets of criteria are commonly used to define and classify TLS: the Cairo‐Bishop and Howard criteria. The Cairo‐Bishop criteria require two or more defined laboratory abnormalities with a 25% change from baseline to occur at any time within 3 days before or 7 days after chemotherapy (Table 1) [11]. Howard et al. proposed that a 25% change from baseline may not be clinically important unless baseline values are already outside of the reference range, and that patients could have one laboratory abnormality and later present with a second abnormality that is actually unrelated to TLS within the time defined by Cairo‐Bishop [12]. Thus, Howard and colleagues modified Cairo‐Bishop criteria by omitting the need for a 25% change in laboratory values and added that the two or more defined laboratory abnormalities (based on absolute values) must present within a 24‐hour period to meet the definition of laboratory TLS [12]. Having laboratory TLS plus an increased creatinine level, seizures, cardiac dysrhythmia, or death constitutes clinical TLS (Table 1).

**Table 1.**
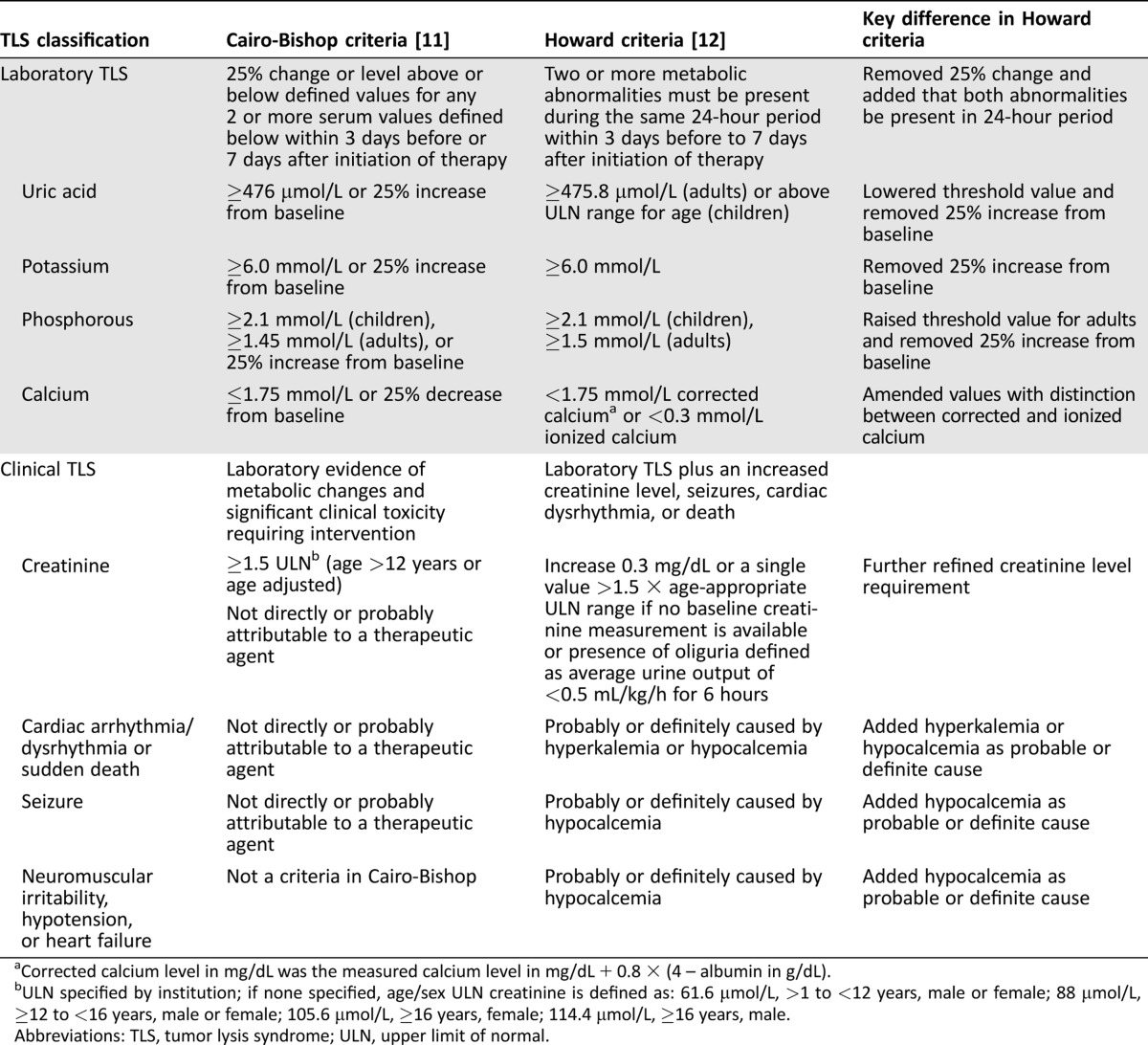
The Cairo‐Bishop and Howard et al. criteria for classification of laboratory and clinical TLS

a Corrected calcium level in mg/dL was the measured calcium level in mg/dL + 0.8 × (4 – albumin in g/dL).

b ULN specified by institution; if none specified, age/sex ULN creatinine is defined as: 61.6 μmol/L, >1 to <12 years, male or female; 88 μmol/L, ≥12 to <16 years, male or female; 105.6 μmol/L, ≥16 years, female; 114.4 μmol/L, ≥16 years, male.

Abbreviations: TLS, tumor lysis syndrome; ULN, upper limit of normal.

## How to Treat TLS: Prevention and Management

Assessing patient risk and anticipating TLS when initiating highly effective therapies is key for prevention of clinically relevant TLS [1], [13]. Approaches to preventing TLS include laboratory monitoring, use of uric acid‐reducing drugs, and adequate hydration, and hospitalization should be considered for patients at high risk. Protocols to monitor urine output and fluid balance, electrolytes (potassium, inorganic phosphorus, calcium), creatinine, and uric acid during initial dosing of CLL treatment enable prompt identification and correction of abnormalities in laboratory findings [12]. A number of features factor into the risk of TLS in individual patients: high tumor burden and/or increased tumor turnover enhances the risk of TLS. However, different therapies may be associated with varying risk of TLS owing to tumor sensitivity to different mechanisms of action (e.g., risk of TLS with venetoclax could be higher than with idelalisib or rituximab). Close laboratory monitoring during the period of risk is highly recommended. General guidelines based on tumor burden as well as other factors (e.g., pre‐existing chronic renal insufficiency, oliguria, splenomegaly, dehydration, hypotension, and acidic urine) recommend that patients at high risk of TLS be monitored every 4–6 hours, that those at medium risk be monitored every 8–12 hours after initiation of therapy, and that low‐risk patients be monitored daily [12]. Current available guidelines are based on extensive experience with chemotherapy, and the general recommendation is that patients should continue to be monitored for at least 24 hours after completion of chemotherapy, as long as electrolyte abnormalities have returned (with or without intervention) to normal [14]. Similar monitoring with new targeted agents is important; in particular, during the 5‐week ramp‐up of venetoclax, monitoring laboratory values at 6–8 hours and 24 hours post each first new weekly dose level (20, 50, 100, 200, and 400 mg daily for weeks 1 through 5, respectively) is recommended [15].

Adequate hydration is critical in the prevention of TLS to promote excretion of uric acid and phosphate. Laboratory TLS can be managed as an outpatient with fluids and an oral hypouricemic agent. Oral hydration may be suitable for patients at low risk and some at medium risk of TLS. However, intravenous fluids are essential for patients at high risk and should be considered for some patients at medium risk of TLS. Aggressive hydration should be administered prior to therapy with the goal of a urine output of at least 100 mL per hour. Unless there is complete loss of kidney function, patients should receive aggressive intravenous fluids to achieve adequate fluid volume for sufficient renal output that should be maintained for 2 days prior to treatment, if possible, and 2–3 days following treatment [10]. Prompt management of hyperkalemia is also crucial to prevention [12], [16]. Diuresis is rarely indicated and may be harmful in the setting of TLS. Thiazide diuretics may increase levels of uric acid and should be avoided. In patients with a low urine output, careful administration of furosemide may be considered. Furosemide can also be used for the management of TLS to prevent severe fluid overload in patients receiving aggressive intravenous hydration [11], [12], [17].

Careful management of electrolytes is critical [14]. There is generally no need to treat for asymptomatic hypocalcemia because of the risk of precipitation of calcium in the kidneys. Hyperphosphatemia can often be managed with phosphate binders, and lowering phosphate levels will help with urate control; however, severe hyperphosphatemia may require dialysis.

Oral hypouricemic agents, including allopurinol or newer agents such as the nonpurine xanthine oxidase inhibitor febuxostat, can be effective prophylactic measures for TLS prevention [18]. Allopurinol is usually given at a fixed dose of 300 mg daily, with febuxostat at 120 mg daily. As allopurinol inhibits uric acid formation, it can take several days to normalize uric acid levels in patients with TLS. Recent data suggest improved prevention of TLS using febuxostat compared with allopurinol in patients with intermediate to high risk for TLS [19]. The National Comprehensive Cancer Network (NCCN) recommends administering allopurinol 2–3 days prior to chemotherapy, with continued treatment for 10–14 days [4].

The NCCN recommends rasburicase for patients with any high‐risk feature (e.g., bulky disease requiring immediate therapy) in whom adequate hydration is not possible or allopurinol is ineffective, or in patients with acute renal failure [4]. Although the schedule of rasburicase was initially recommended as 0.2 mg/kg daily for up to 5 days, subsequent data support a single dose of 0.15–0.2 mg/kg, with a second dose the next day if the uric acid level is not back to within normal range [20]. A single fixed dose of 6 mg rasburicase administered intravenously is also common for lowering uric acid in the management of TLS [21], [22]. Although rasburicase is effective in correcting hyperuricemia, there are no randomized controlled trials showing that it prevents renal failure or death [23], [24]. Patients who are considered candidates for rasburicase, especially those of African or Mediterranean descent, should first be tested for glucose‐6‐phosphate dehydrogenase deficiency because those with a deficiency of the enzyme are at risk for hemolysis and methemoglobinemia.

Alkalinization of the urine is no longer recommended as a management strategy because it may be associated with metabolic acidosis and calcium phosphate precipitation [1], [25]. To avoid the risk of nephropathy due to calcium phosphate deposition, treatment of asymptomatic hypocalcemia is generally not recommended. Dialysis should be considered for patients with acute kidney injury who have life‐threatening electrolyte disturbances [1].

## TLS Experience with Newer CLL Therapies

Although historically observed but not severe [9], TLS is now more frequently noted with some of the newer agents approved for treatment of CLL (Table 2). The rate of TLS with approved anti‐CD20 monoclonal antibodies, including rituximab, obinutuzumab, and ofatumumab, used as monotherapy or in combination with other agents for treatment of CLL, has been low [7], [26], [27], [28], [29], [30], [31], [32], [33], [34], [35], [36]. A high frequency of biochemical TLS, as well as a number of cases of hyperacute TLS defined as tumor lysis requiring dialysis within 6 hours of initiating therapy, have been observed with the serine/threonine kinase inhibitor Alvocidib (flavopiridol; Tolero Pharmaceuticals, Lehi, UT, http://www.toleropharma.com) [37]. Of 116 patients in a retrospective analysis [38], the incidence of TLS was 46%. Female sex, greater number of prior therapies, Rai stages III and IV, bulky adenopathy, splenomegaly, increased absolute lymphocyte count, white blood cell count, β_2_‐microglobulin level, and decreased albumin all correlated with TLS. The occurrence of TLS did not appear to predict treatment response. Tumor lysis syndrome still occurred in a subsequent phase 1 study using a modified dose and dosing schedule and correlated with drug metabolite exposure and white blood cell count greater than 200 × 10^9^/L [39].

**Table 2.**
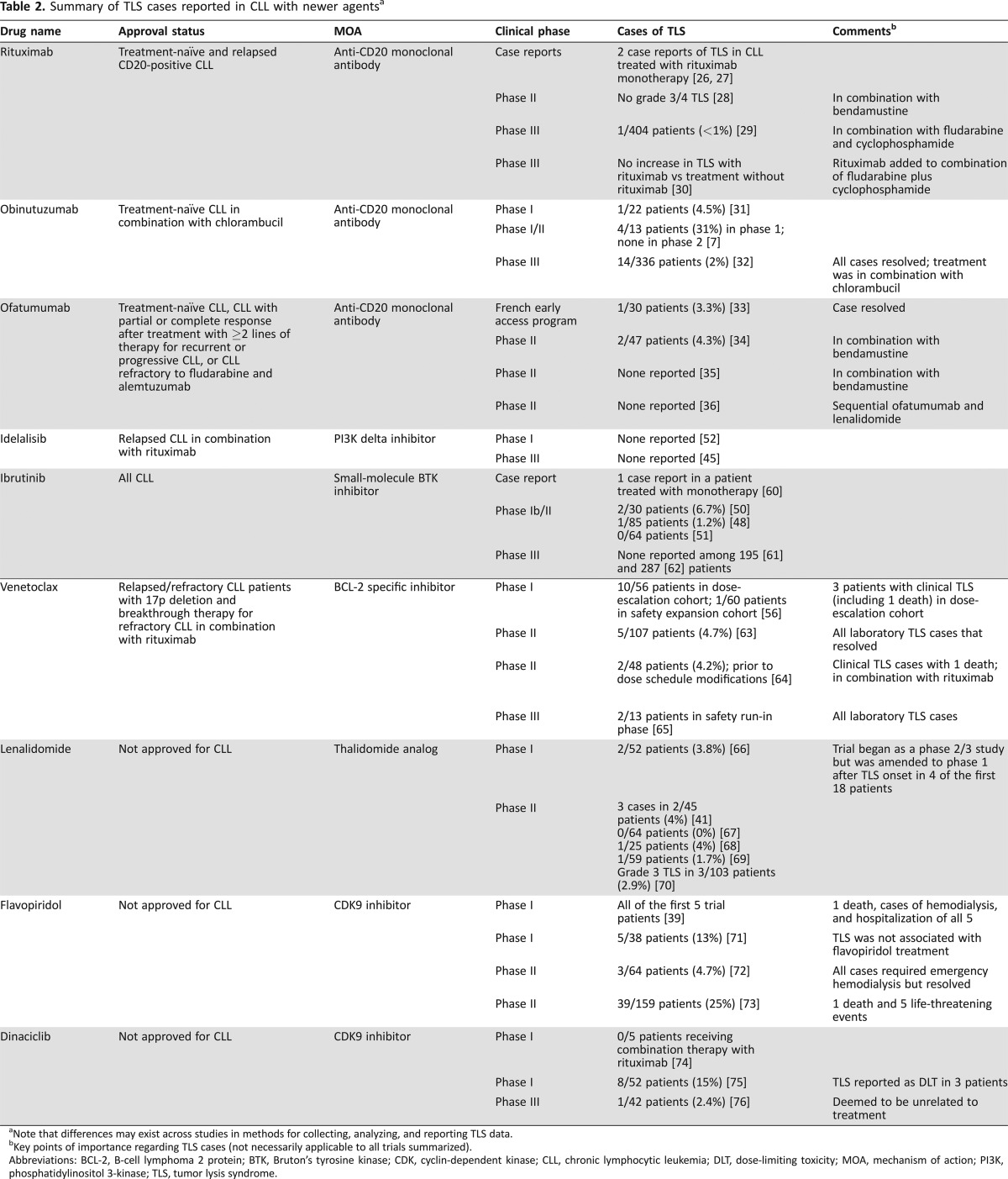
Summary of TLS cases reported in CLL with newer agents^a^

a Note that differences may exist across studies in methods for collecting, analyzing, and reporting TLS data.

b Key points of importance regarding TLS cases (not necessarily applicable to all trials summarized).

Abbreviations: BCL‐2, B‐cell lymphoma 2 protein; BTK, Bruton's tyrosine kinase; CDK, cyclin‐dependent kinase; CLL, chronic lymphocytic leukemia; DLT, dose‐limiting toxicity; MOA, mechanism of action; PI3K, phosphatidylinositol 3‐kinase; TLS, tumor lysis syndrome.

Lenalidomide has modest activity as a single agent in CLL, but has also been associated with tumor flare and TLS [40]. In a phase 2 study by Chanan‐Khan et al. [41], of 45 patients, 2 developed TLS, both during the first cycle. In one patient, TLS recurred in the second cycle despite a reduced dose of lenalidomide.

The B‐cell receptor pathway inhibitors ibrutinib and idelalisib have demonstrated a high level of efficacy in patients with CLL [42], [43]. Despite the fact that these drugs result in a very rapid decrease in node size, often accompanied by a transient lymphocytosis as a result of demargination, TLS is exceptionally uncommon [44], [45], [46], [47], [48], [49]. The package insert for ibrutinib lists TLS risk as a potential serious adverse event because of a few cases described in phase 2 studies, despite a low overall risk [42], [48], [50], [51]. No cases of TLS have been reported with idelalisib [45], [52]. Given the infrequency of this observation with kinase inhibitors, it is not possible to identify risk factors from the available data. The recent enthusiasm for chimeric antigen receptor‐modified T cells is associated with a recognition of potentially life‐threatening adverse effects, including TLS [53], [54].

Venetoclax is a highly selective oral inhibitor of the antiapoptotic B‐cell lymphoma‐2 protein. Two fatalities from TLS were reported in early studies using a dosing strategy starting at 50 mg daily venetoclax followed by a 3‐week ramp‐up to a maximum dose of 1,200 mg. These cases prompted refinement of venetoclax dosing and TLS mitigation strategies, which are described in the section “TLS with Venetoclax: A Clinical Example.” Overall, the rate of TLS is low with newer agents; however, because TLS can potentially be fatal, understanding of individual risk and potential strategies to minimize its occurrence is critical for effective management.

### TLS with Venetoclax: A Clinical Example

**Figure 1.**
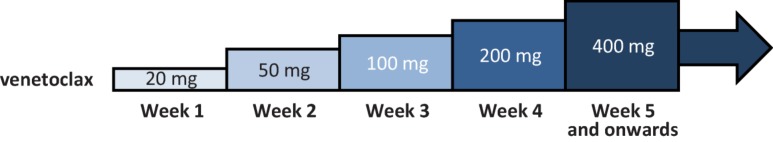
Final recommended once‐daily dosing schedule for venetoclax 5‐week dose ramp‐up used in clinical trials for patients with chronic lymphocytic leukemia and/or small lymphocytic lymphoma.

Venetoclax is now approved for patients with CLL that harbors the chromosome 17p deletion who received at least one prior therapy, or for patients without the 17p deletion after at least one prior therapy and for whom there are no other available therapies [15], [55]. Venetoclax achieves high response rates in patients with relapsed/refractory CLL, and data from the first‐in‐human clinical study showed rapid response to treatment [56]. In the initial cohort (*n* = 56) of this first‐in‐human study with venetoclax, three patients experienced clinical TLS and seven had laboratory TLS (based on Howard criteria; Table 1), even with dose ramp‐up to allow for gradual tumor reduction (i.e., venetoclax initiated at 50 mg and ramped up to a maximum target dose of 1,200 mg over 3 weeks) and TLS prophylaxis. Of the three patients with clinical TLS, one died suddenly after ramping up to 1,200 mg daily, one died following acute hyperkalemia at a dose of 50 mg, and one experienced acute renal failure requiring dialysis after an initial 50 mg dose [56]. Based on these reports of TLS, dosing was amended such that venetoclax was initiated with a lower 20 mg dose for 1 week, followed by gradual ramp‐up over 5 weeks to a target dose of 400 mg (recommended phase 2 dose of venetoclax; Fig. 1) along with intensive inpatient prophylaxis [57], [58]. Subsequently, 60 patients were enrolled in the expansion cohort of the first‐in‐human study; they received venetoclax single agent based on this modified dosing and gradual weekly ramp‐up schedule, and one of the 60 patients was reported to have laboratory evidence of TLS that resolved without clinical sequelae, with no instances of clinical TLS [56].

A comprehensive data review of reports of TLS across venetoclax clinical trials revealed that a combination of tumor burden (bulky lymph nodes ≥5 cm and/or elevated absolute lymphocyte count ≥25 × 10^9^) and reduced renal function at screening could be used to identify patients at risk of developing TLS, and guidelines per risk category were implemented [57]. Along with the gradual 5‐week dose ramp‐up described above (Fig. 1), all patients now receive oral or intravenous fluids, based on the risk of TLS, beginning 48 hours prior to the first dose of venetoclax, as well as oral uric acid‐reducing agents starting 72 hours prior to first dose. Rasburicase is recommended for patients at high risk for developing TLS and who have high baseline uric acid levels. For patients with low to medium tumor burden, dosing can be in an outpatient setting, with monitoring of laboratory values within 72 hours prior to the first dose at each step of the ramp‐up, and at 0, 8, and 24 hours after dose (Table 3). For patients with high tumor burden or those with creatinine clearance <80 mL per minute, the initial 20 mg and 50 mg doses are given in the hospital, with laboratory monitoring at 0, 4, 8, 12, and 24 hours; subsequent ramp‐up doses may be given in an outpatient setting (Table 3). Based on clinical data from venetoclax studies, up to 24 hours is the period of time that requires monitoring for risk of TLS when initiating therapy. With these current measures, four of 296 patients across three clinical studies of venetoclax monotherapy had five adverse events of TLS as reported by investigators (one patient had two events) [59]. Based on medical review, no clinical TLS was observed and only one adverse event met Howard criteria for laboratory TLS (decreased calcium and increased phosphate). All of the laboratory TLS events were observed within the 5‐week dose ramp‐up and were managed with prompt hydration, electrolyte correction, and temporary dose interruption. Patients resumed dosing without clinical sequelae and no patient discontinued venetoclax owing to TLS [59].

**Table 3.**
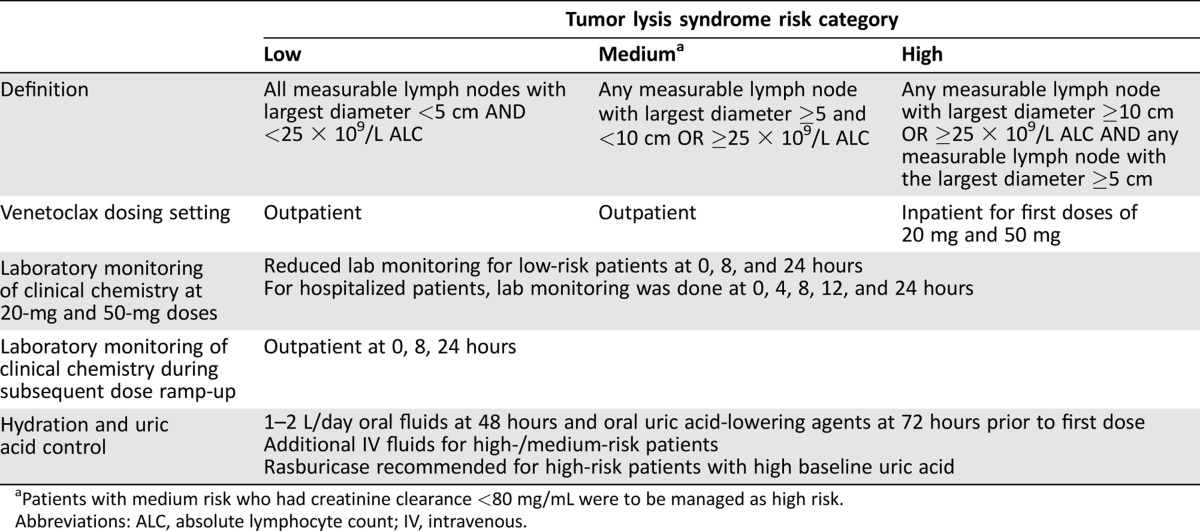
Venetoclax prophylaxis and monitoring approach for patients with relapsed/refractory chronic lymphocytic leukemia/small lymphocytic lymphoma [57], [58]

a Patients with medium risk who had creatinine clearance <80 mg/mL were to be managed as high risk.

Abbreviations: ALC, absolute lymphocyte count; IV, intravenous.

From early clinical development, the modification to venetoclax dosing as well as recommended prophylaxis and monitoring based on risk for TLS have largely reduced the frequency and severity of TLS, particularly clinical TLS, seen with venetoclax. This approach may also be an option for limiting TLS risk with other highly effective targeted agents or with new combination therapies.

A comprehensive data review of reports of TLS across venetoclax clinical trials revealed that a combination of tumor burden (bulky lymph nodes ≥5 cm and/or elevated absolute lymphocyte count ≥25 × 10^9^) and reduced renal function at screening could be used to identify patients at risk of developing TLS, and guidelines per risk category were implemented.

## Conclusion

Because we are now in an era of more effective therapies for CLL, TLS must be anticipated, especially for patients determined as being at high risk. The venetoclax clinical development program provides an example in which principles of risk management were systematically applied to allow for mitigation of TLS risk and continued use of the therapy. Although the overall incidence of TLS is low, optimal patient management is critical to mitigate even the possibility of a fatal event. The possibility of reducing tumor burden with cytotoxic drugs or other biological agents (e.g., monoclonal antibodies, kinase inhibitors) prior to venetoclax therapy, in order to reduce the likelihood of TLS, is now being studied in clinical trials (e.g., NCT02401503 and NCT02427451). However, using the new schedule of venetoclax administration, TLS has become a less common occurrence. With proper dosing and adherence to prophylaxis and monitoring guidelines appropriate for the level of risk of TLS at baseline, current and future CLL therapies can be safely administered with effective disease control.
